# Co‐occurrence of pederin‐producing and *Wolbachia *endobacteria in *Paederus fuscipes* Curtis, 1840 (Coleoptera: Staphilinidae) and its evolutionary consequences

**DOI:** 10.1002/mbo3.777

**Published:** 2018-12-17

**Authors:** Naseh Maleki‐Ravasan, Niloofar Akhavan, Abbasali Raz, Mahmood Jafari, Sedigheh Zakeri, Navid Dinparast Djadid

**Affiliations:** ^1^ Malaria and Vector Research Group (MVRG), Biotechnology Research Center (BRC) Pasteur Institute of Iran Tehran Iran; ^2^ Department of Biotechnology, Faculty of Advanced Sciences and Technology, Pharmaceutical Sciences Branch Islamic Azad University Tehran Iran; ^3^ Department of Geology, Faculty of Sciences Tarbiat Modares University Tehran Iran

**Keywords:** *16S rRNA*, *OprF*, *Paederus*, pederin, *Pseudomonas*‐like, *wsp*

## Abstract

The dual occurrence of *Pseudomonas*‐like and *Wolbachia* endobacteria has not been investigated in the *Pederus* beetles yet. We investigated pederin‐producing bacteria (PPB) infection in *Paederus fuscipes *specimens from the southern margins of the Caspian Sea by designed genus‐specific (*OprF*) and species‐specific (*16S rRNA*) primers. *Wolbachia* infection was studied through a nested‐PCR assay of *Wolbachia* surface protein (*wsp*) gene. Of the 125 analyzed beetles, 42 females (82.35%) and 15 males (20.27%) were positive to PPB infection; this is the first study reporting male *P. fuscipes* infection to PPB. *Wolbachia* infection was found in 45 female (88.23%) and 50 male (67.57%) analyzed beetles. Surprisingly, a number of 36 females (70.59%) and 13 males (17.57%) were found to be infected with both PPB and *Wolbachia *endosymbionts. In general, population infection rates to PPB and *Wolbachia* were determined to be 45.6% and 76%, respectively. The infection rates of female beetles to PPB and PPB‐*Wolbachia* were significantly higher than males. In *Paederus* species, only female beetles shelter PPB and the discovery of this bacterium in adult males may reflect their cannibalistic behavior on the contaminated stages. Phylogenetic analysis showed that the sequences of *OprF *gene were unique among *Pseudomonas* spp.; however, sequences of *16S rRNA* gene were related to the PPB of *Pederus* species. The co‐occurrence and random distribution of these endobacteria may imply putative tripartite interactions among PPB, *Wolbachia*, and *Paederus*. In order to elucidate these possible tripartite interactions, further studies are required even at gender level.

## 1 INTRODUCTION

Rove beetles of Staphylinidae are the largest family of beetles and are distributed in a wide range of habitats. They include more than 63,000 known species arranged into thousands of genera and 32 subfamilies worldwide (Grebennikov & Newton, 2009; Thayer, 2005). The genus *Paederus* Fabricius, 1775, which is classified into the tribe Paederini and subfamily Paederinae, currently comprises ~490 species (Nikbakhtzadeh, Naderi, & Safa, 2012; Vieira, Ribeiro‐Costa, & Caron, 2014).

Fourteen species and subspecies of the *Paederus*, including five subgenera occur in Iran. Among them, six species *P. balachowskyi, P. balcanicus, P. duplex, P. fuscipes fuscipes, P. littoralis ilsae*, and *P. riparius *are present in three Southern Caspian Provinces, Gilan, Mazandaran, and Golestan (Nikbakhtzadeh et al., 2012).

In natural ecosystems (predominantly moist environments), staphylinidaes are connected with various arthropods, higher plants, fungi, decomposing materials, mollusks, and vertebrates. Most of the rove beetles are predators of arthropods, and some of them are associated with social insects, while others are scavengers on decaying plant matter or live in nests of rodents (Frank & Thomas, 2016). Some species of rove beetles are important in terms of the biological control of insects of agricultural, medical, and veterinary importance (Echegaray & Cloyd, 2013).

*Paederus* species and its relatives are the agents of human dermatitis as well. They are active during daylight hours and can cause linear dermatitis on human skin and severe damage to human eyes (Nairobi eye). These beetles neither bite nor sting but release their hemolymph containing pederin, a potent vesicant toxin (C_25_H_45_NO_9_; MM: 503.63; LD50: 0.14 mg/kg rat i.p.), when they injured or crushed on human skin (Dettner, 2011; Iserson & Walton, 2012). This contact dermatitis is a distinctive stimulus form that can be distinguished by the rapid onset of erythematobullous lesions on the exposed areas (Mammino, 2011). If erythemas continue longer, other symptoms such as fever, edema, neuralgia, arthralgia, and vomiting may be observed as well (Rahman, 2006). It is supposed that pederin has antitumor and antiviral properties (Narquizian & Kocienski, 2000), presumably through the inhibition of DNA replication and protein synthesis (Dettner, 2011).

Pederin and its derivatives, namely, pseudoephedrine and pederone, are synthesized by uncultured *Pseudomonas*‐like endosymbionts located in the female accessory glands, restored in the hemolymph and transferred to the developmental stages through the contaminated eggs (Kellner, 1998, 2001; Kellner & Dettner, 1995). Studies based on *16S rRNA* gene have shown that only female beetles contain the pederin‐producing bacteria (PPB; Kellner, 2002). These bacteria are distributed in the rove beetle populations, through the transovarial transmission (Kador, Horn, & Dettner, 2011).

Naturally, pederin is used by *Paederus* species as a defensive compound against insect and arachnid predators (Kellner & Dettner, 1996). The immature stages of *P. fuscipes* and *P. riparius*, which were pederin positive, were repulsive for spiders of the families Lycosidae and Salticidae but not for insect predators (Kellner & Dettner, 1996).

*Wolbachia*, obligate endosymbionts, are estimated to infect 40% of terrestrial arthropod species (Zug & Hammerstein, 2012) and many parasitic filarial nematodes (Taylor, Bandi, & Hoerauf, 2005). They manipulate reproduction properties of the hosts through the induction of cytoplasmic incompatibility, parthenogenesis, feminization, and male killing (Hughes, Pamilo, & Kathirithamby, 2004; Li et al., 2016; Li, Wang, Bourguet, & He, 2013; Vavre, Fleury, Lepetit, Fouillet, & Boulétreau, 1999; Werren, 1997; Yun, Peng, Liu, & Lei, 2011).

*Wolbachia* strains and their role in arthropod host fitness have been reviewed recently (Zug & Hammerstein, 2015). It has been indicated that a *Wolbachia* strain could protect alfalfa weevil, *Hypera postica,* against the parasitic wasp, *Microctonus aethiopoides* (Hsiao, 1996). Recently, it has been shown that *Wolbachia* can protect *Culex pipiens* mosquitoes against *Plasmodium relictum*‐induced mortality (Zele, Nicot, Duron, & Rivero, 2012). In addition, a new strain of *Wolbachia* has been reported in *Cimex lectularius* that appears to display an important role in bedbug fitness through provisioning of B vitamins (Nikoh et al., 2014). More recently, some strains of *Wolbachia* have been introduced as a weapon in the war against vector‐borne pathogens (Hughes & Rasgon, 2014; Kambris, Cook, Phuc, & Sinkins, 2009). Therefore, a variety of *Wolbachia* strains can have either mutualistic or parasitic outcomes in the insect/pathogens/parasitoids assemblages (van Nouhuys, Kohonen, & Duplouy, 2016), which should be studied in details when their properties are exploited.

Initially, insect's isolates of *Wolbachia pipientis* has been classified into two supergroups (A and B) and 12 groups based on the sequences of the major *Wolbachia* surface protein (*wsp*) gene (Zhou, Rousset, & O'Neill, 1998). Today, all invertebrate isolates of *Wolbachia* have been divided sequentially into 16 supergroups (A to Q) using the multilocus sequence typing (MLST) technique (Baldo et al., 2006; Glowska, Dragun‐Damian, Dabert, & Gerth, 2015).

Despite many advances in the study on *Wolbachia* infection in insects, our knowledge on the *Wolbachia* strain diversity/dispersion, or their effects on the beetle hosts is very limited. According to the findings of a review study (Kajtoch & Kotásková, 2018), *Wolbachia* infection was detected in 204 coleopteran species with average prevalence of 38.3%. The most intensively studied families have been herbivorous beetles of Curculionidae and Chrysomelidae. Coleoptera‐infecting *Wolbachia* strains belonged to three supergroups of A, B, and F with single, double, or multiple infections in the studied species. *Wolbachia* has had a lot of effects on its beetle hosts ranging from selective sweep with host mtDNA and cytoplasmic incompatibility to other changes related to the reproductive or developmental phenotypes (Kajtoch & Kotásková, 2018).

Survival and reproduction of many insects rely on the endosymbiotic bacteria (Eleftherianos, Atri, Accetta, & Castillo, 2013; Ratzka, Gross, & Feldhaar, 2012). Therefore, PPB as defensive (Oliver & Moran, 2009) and *Wolbachia* as reproductive (Kajtoch & Kotásková, 2018) symbionts may play an important role in evolution and adaptation of *Paederus* species. As a matter of fact, PPB seems to affect the capacity of the *Paederus* beetles to be causative agent of human linear dermatitis. It is also necessary to study the distribution of *Wolbachia* in the rove beetles to determine its function in host biology. Infection of *Paederus *species by PPB and *Wolbachia* has separately been investigated in a very few studies (Kador et al., 2011; Yun et al., 2011); however, dual occurring of these endobacteria has not been investigated yet. Hence, we studied co‐occurrence of PPB and *Wolbachia *in *P. fuscipes*. The achieved results can contribute to pave the way to address interesting open queries on the evolutionary consequences of the interactions between these inherited bacteria and their host biology with further experiments.

## 2 EXPERIMENTAL PROCEDURES

### 2.1 Study areas

The specimens were collected from nine locations of two provinces of southern coast of Caspian Sea in Iran, Gilan (Bijar Boneh [*n* = 6], Vashmeh Sara [*n* = 38], Kochesfehan [*n* = 10], Chini Jan [*n* = 8], Kalachai [*n* = 1], and Tajan Gukeh [*n* = 40]) and Mazandaran (Royan [*n* = 6], Shirud [*n* = 5], and Amol [*n* = 11]). Live adult beetles were gathered from humid areas, principally from rice fields, using hand collection method. The specimens were kept in 70% ethanol in 4°C refrigerator until experiments.

### 2.2 Morphological studies

The specimens were morphologically identified using available identification keys generated by Blackwelder (1957), Arnett and Thomas (2001), and Borror and DeLong's (Triplehorn & Johnson, 2005).

### 2.3 DNA extraction

Prior to molecular survey, to surface sterilize, the specimens were immersed twice in freshly prepared 70% ethanol for 2 min and rinsed vigorously with 0.9% normal saline. The whole bodies of adult beetles were homogenized in the DNA lysis buffer using sterile pestles. Genomic DNA of rove beetles was extracted using Collins DNA extraction method (Collins et al., 1987).

### 2.4 Detection of PPB infection

#### 2.4.1 *OprF* primer designing and amplification

The major outer membrane protein of *Pseudomonas*, *OprF*, has been found only in *Pseudomonas* genus and considered as a diagnostic protein in *Pseudomonas *sensu stricto (Bodilis & Barray, 2006; Bouffartigues et al., 2011). A total of 44 sequences of *OprF* gene related to *Pseudomonas* isolates were extracted from the GenBank and aligned using Mega 5.0 software. The conserved regions among all *Pseudomonas *isolates were targeted to design genus‐specific primers. Two primers, OPRFF: 5'‐GTGGA(A/G)GTGGACGGGTACTGCTTCATG‐3' and OPRFR: 5'‐CAACGGTCACCAGGGCGAGTGGATG‐3', were designed based on the *OprF*‐specific sequences to amplify 327 bp of *Pseudomonas* spp. and PPB in the rove beetles. PCRs were done in a volume of 20 μl containing 5 pmol of each designed primer (Macrogen, Korea), 0.5 nmol dNTPs (Fermentas, USA), 1 U Taq DNA polymerase (CinnaGen, Iran), 2.5 μl buffer 10×, and 1–5 μl (~0.1 μg) of the extracted DNA from samples. The PCR thermal profile used with these primers was an initial denaturation at 95°C for 5 min, followed by 35 cycles of 94°C for 30 s, 66°C for 30 s, 72°C for 25 s, and a final extension step at 72°C for 10 min. All specimens were firstly screened with *OprF* gene, and then positive ones were examined via *16S rRNA* gene.

#### 2.4.2 *16S rRNA* primer designing and amplification

Five available *16S rRNA* sequences of PPB in rove beetles (*P. fuscipes* [AJ316016], *P. riparius* [AJ316018], *P. melanurus* [AJ316017], *P. ruficollis* [AJ316019], and *P. sabaeus* [AJ295331]) and five representative *16S rRNA* sequences of other bacteria (*Pseudomonas aeruginosa* [AE004844], *Escherichia fergusonii* [NR_074902], *Salmonella enteric* [NR_119108], *Klebsiella pneumoniae* [NR_117686], and *Proteus mirabilis* [NR_114419]) were retrieved from the GenBank and subjected to PPB species‐specific primer designing. After alignment, 16S‐PPBF: 5'‐ACCGCATACGTCCTAAGGGAG‐3' and 16S‐PPBR: 5'‐CCTCCTTGCGGTTAGACCAG‐3’ primers were designed based on the *16S rRNA*‐specific sequences of PPB in rove beetles to amplify a 1,265‐bp fragment of this gene. PCRs were the same as those performed for *OprF* primers. After an initial denaturation step of 5 min at 94°C, 35 cycles were carried out (denaturation for 30 s at 94°C, annealing for 30 s at 59°C, and elongation for 80 s at 72°C), followed by 10 min at 72°C.

### 2.5 Detection of *Wolbachia* infection

*Wolbachia* infection was detected in rove beetles on the basis of Zhou et al.'s, (1998) introduced primers and through a nested‐PCR assay recruited by Karami et al., (2016). Originally, primers of 81F: 5'‐TGGTCCAATAAGTGATGAAGAAAC‐3' and 691R: 5'‐ AAAAATTAAACGCTACTCCA‐3' were applied to amplify a 632‐bp of partial sequence of the *wsp* gene. The PCR product of the first step was employed as a template for the second step. In this step, the primers of 691R and 183F: 5'‐AAGGAACCGAAGTTCATG‐3' were used to amplify a 501‐bp fragment. The PCR was performed in a total volume of 20 μl containing 5 μl (~0.5 μg) of genomic DNA for the first step, and 2.5 μl of PCR product for the second step of nested‐PCR, one‐time PCR buffer, 2.5 U Taq polymerase (CinnaGen, Iran), 1 μl of each primer (20 mM, Macrogen, Korea), 200 μM of each dATP, dTTP, dCTP, and dGTP (Fermentas, USA) and 1.5 mM of MgCl_2_ in an automated Thermocycler (Analytik Jena FlexCycler, Canada). The PCR conditions were set as an initial denaturation at 95°C for 5 min, followed by 35 cycles of denaturation at 94°C for 1 min, annealing at 55°C for 1 min, and extension at 72°C for 1 min, followed by a final extension at 72°C for 7 min.

### 2.6 Sequencing and phylogenetic analysis

All the PCR products from *16s rRNA*, *OprF*, and *wsp* genes were analyzed by 1% agarose gel electrophoresis, followed by Green Viewer staining and visualization using a UV transilluminator. Amplicons of the representative specimens were extracted from the gel, and after purification was sequenced bidirectionally via the same amplification primers (Macrogen Company, Korea).

The raw sequences were initially edited by the Chromas 2.6.5 software through trimming of right and left cut‐off regions that may contain poor qualities. The consensus of confident sequences was analyzed using NCBI (nucleotide collection) database. Multiple alignments of the studied sequences were generated by the Clustal Omega package (Sievers et al., 2011). BLOSUM62 and Kimura‐2‐Parameter models were used to, respectively, score the pairs of aligned *OprF*/*wsp* amino acids and *16S rRNA* nucleotides. Phylogenetic trees were constructed using the maximum likelihood method embedded in Mega 5 software (Tamura et al., 2011). Confidence of internal nodes was tested by Bootstrap test with 1,000 replications.

The phylogeny of various *Pseudomonas *spp., including PPB, was evaluated based on the *OprF* gene sequences. The relationships between *16S rRNA* gene sequences of PPB in *Paederus* specimens and their close relative, *Pseudomonas aeruginosa,* was investigated through the phylogenetic tree construction.

### 2.7 Statistical analysis

SPSS 20 for windows (SPSS Inc., USA) was used for statistical analysis. Differences between the proportions of subjects positive for each one of the *Wolbachia* and BBP bacteria or their combination in females and males were assessed using Chi‐square (χ^2^) test. *p* values <0.05 were considered statistically significant.

## 3 RESULTS

### 3.1 Morphological study

In this research, a total of 125 adult rove beetles, including 74 males and 51 females, were studied. All the collected specimens were taxonomically identified as *Paederus fuscipes* Curtis, 1840 (Coleoptera: Staphilinidae) by using the morphological keys mentioned in the Experimental Procedures.

### 3.2 Detection of PPB and *Wolbachia* infection in *P. fuscipes*

Prior to practical procedures, the specificity of designed primers was tested in silico. Performing BLAST searches showed that *OprF* primers were able to find cultured and uncultured *Pseudomonas* spp., which is in accordance with the desired specificity we expect for our study to identify *Pseudomonas* and *Pseudomonas*‐like species, but not the other genera. Also, the *16S rRNA* primers could amplify only PPB endosymbiont of *P. fuscipes*, and it did not even reproduce symbionts which were present in the GenBank other than *P. fuscipes*.

In practice, both *Pseudomonas*‐specific (*OprF*) and PPB‐specific (*16S rRNA*) primers resulted in amplicon sizes of 327 and 1,265 bp, respectively, as expected. Applying the nested‐PCR assay could easily detect the *wsp*, a single‐copy gene. The PCR products of the first and the second steps of nested‐PCR assay were roughly 650 and 500 bp, respectively.

### 3.3 PPB and *Wolbachia* infection rates in *P. fuscipes*

**Figure 1:**
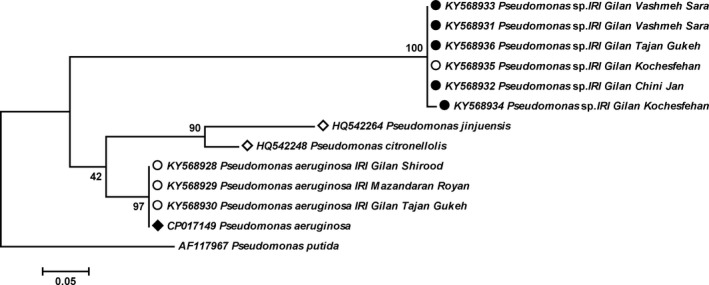
Maximum likelihood tree showing the phylogenetic relationships between the OprF gene sequences obtained in this study (solid/empty circles) and other isolates of *Pseudomonas* spp. Solid and empty circles: bacterial genome amplified from female and male *Paederus fuscipes*, respectively; solid diamond: clinical isolate of *Pseudomonas* spp.; empty diamonds: environmental isolate of *Pseudomonas* spp. *Pseudomonas putida* was designated as outgroup. The numbers at the branch points are bootstrap values based on 1,000 replicates

**Figure 2:**
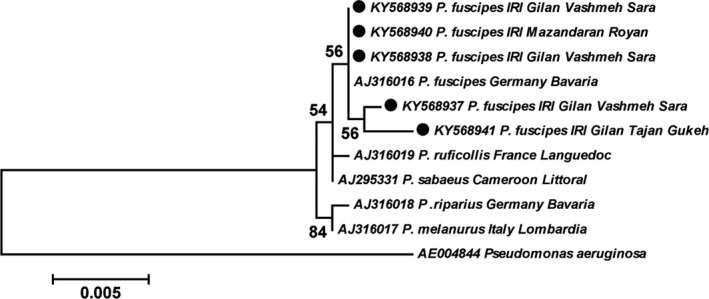
Maximum likelihood tree showing the phylogenetic relationships among pederin‐producing bacteria (PPB) in *Paederus* spp. based on 16S rRNA gene sequences. Sequences obtained in this study are shown by solid circles. *Pseudomonas aeruginosa* was set as outgroup. The numbers at the branch points are bootstrap values based on 1,000 replicates

The designed *OprF* primers could amplify all *Pseudomonas* species, including *Pseudomonas*‐like PPB and *P. aeruginosa* (Figure 1). However, the species‐specific *16S rRNA* primers that we designed could identify only *Pseudomonas*‐like PPB (Figure 2). In total, of the 125 (51 female and 74 male) analyzed beetles, 42 females (82.35%) and 15 males (20.27%) were positive to *OprF* primers and the same rates (82.35% and 20.27%) were also positive to the PPB‐specific *16S rRNA* primers. PPB was detected not only in female beetles (as reported by Kellner, 2002) but also in male beetles. This is the first study reporting male *P. fuscipes* infection to PPB. Also, *Wolbachia* infection was found in 45 female (88.23%) and 50 male (67.57%) analyzed beetles. Surprisingly, a number of 36 females (70.59%) and 13 males (17.57%) were detected to be infected with both PPB and *Wolbachia* endosymbionts. Individual analysis of bacteria showed that six females (11.76%) and two males (2.7%) were PPB positive and nine females (17.65%) and 37 males (50%) were positive for *wsp* gene.

The Chi‐squared test showed no significant difference (*p* = 0.13) of *Wolbachia* infection among male and female beetles, either alone or in combination with *Pseudomonas*. The infection rates of females to PPB and PPB‐*Wolbachia *were significantly higher than males in both alone and combined analyses (χ^2^, *p < 0.05*). Combined analysis showed that *Wolbachia* infection rate in females was more than males; however, this difference was not significant (χ^2^, *p* = 0.015). Overall, our results pointed out that 45.6% and 76% of all the specimens were positive to PPB and *Wolbachia* endosymbionts, respectively. The infection results in both alone and combined analyses are depicted in Table 1.

**Table 1:** Prevalence of PPB and *Wolbachia* infection in the *Paederus fuscipes* specimens collected from nine locations of two Northern provinces of Iran during 2016

Endosymbiont beetle gender	Alone			Combined	
	PPB (%)	*Wolbachia* (%)	PPB‐*Wolbachia* (%)	PPB (%)	*Wolbachia* (%)
Male	2 (2.7)	37 (50)	13 (17.57)	15 (20.27)	50 (67.57)
Female	6 (11.76)	9 (17.65)	36 (70.59)	42 (82.35)	45 (88.23)
Total	8 (6.4)	46 (36.8)	49 (39.2)	57 (45.6)	95 (76)

### 3.4 Sequence and phylogenetic analyses

Sequence analysis of *OprF* gene revealed the presence of two phylogenetically diverse groups in both male and female rove beetles; the first group of PPB sequences had 78% similarity to *P. jinjuensis* and *P. citronellolis*, and the second group of sequences was 100% identical to *P. aeroginosa* (Figure 1). Phylogenetic analysis showed that the sequences of *OprF *gene are unique among *Pseudomonas* spp.; however, the sequences of *16S rRNA* gene were related to the PPB of *Pederus* species.

Comparative *16S rRNA* gene sequence analysis showed that some specimens from Gilan (KY568938 & KY568939) and Mazandaran (KY568940) Provinces were 100% identical to each other. Nevertheless, there were minor differences between the samples from Gilan Province (KY568941 with 4 and KY568937 with 2 substitutions). In general, phylogenetic analysis of *16S rRNA* gene from *P. fuscipes* specimens indicated that along with a sequence of the same species from Germany, the sequences of this study made a monophyletic clade were located as the sister clade of the sequences from *P. ruficollis* (France) and *P. sabaeus* (Cameroon; Figure 2).

The *wsp* gene sequence analysis displayed that all *Wolbachia* strains, obtained from the collected *P. fuscipes* in the study areas, were 100% identical to each other. In addition, the results of the BLAST search indicated that these strains were fully similar to the *wsp* sequence of *Aedes albopictus* [AF020059], *Drosophila simulans* [AF020069 and AF020074], *Culex pipiens* [AF020061], and *Lasioderma serricorne* [AB469359], the members of the *Pip* group of supergroup B.

### 3.5 Nucleotide sequence accession numbers

The nucleotide sequences determined in this study have been deposited into the GenBank database under the following accession numbers; *OprF*: KY568928–KY568936, *16s rRNA*: KY568937–KY568941, and *wsp* gene: KY555600–KY555603. The representatives of each sequences group were applied to phylogenetic analysis (Figures 1 and 2).

## 4 DISCUSSION

We studied dual occurrence of PPB and *Wolbachia* endobacteria in *P. fuscipes* rove beetles. The overall population infection rates to PPB and *Wolbachia* endosymbionts were revealed to be 45.6% and 76%, respectively. The PPB infection has previously been reported only in adult females (Kellner, 2002); however, here, we report the infection not only in females but also in male specimens. Detection of PPB in male beetles does not necessarily mean the existence of pederin substance in the male beetles. The PPB infection in females was found to be four times that of males (Table 1). These results are rational because the female *Paederus* have to transmit PPB to offspring and protect them against both conspecific and other natural predators. Finding the PPB infection in adult males may be reflecting the cannibalistic behavior of the rove beetles, in part.

In this study, for the first time, PPB was detected at the genus and species levels, respectively, by *OprF* and *16S rRNA* primers. The outcoming results from both genus and species levels were in agreement with the detection of PPB. Initially, the specimens were screened with *OprF* gene (copy numbers ≃ 200,000 per bacterial genome [Hancock, Siehnel, & Martin, 1990]), and then positive specimens were examined via *16S rRNA* gene with copy numbers 1–15 per bacterial genome (Rainey, Ward‐Rainey, Janssen, & Hippe, 1996). The *OprF* is a protein that not only has widely been studied in vaccine researches (Rawling, Martin, & Hancock, 1995) but also considered as a diagnostic protein for *Pseudomonas *spp. (Bouffartigues et al., 2011). Our designed *OprF* primers could amplify both *Pseudomonas*‐like PPB and *P. aeruginosa* (Figure 1). The *P. aeruginosa* is extensively distributed in the environment and can be both opportunistic and pathogenic microbial agent of plants, animals, and humans (Balcht & Smith, 1994). It has frequently been isolated from medical and nonmedical insects (Bulla, Rhodes, & St. Julian, G., 1975; Maleki‐Ravasan et al., 2015; Mitscherlich & Marth, 1984). *Pseudomonas* strains found in insects have been shown to protect host against toxic compounds in some cases (e.g., Ceja‐Navarro et al., 2015); however, they display pathogenic characteristics in general (Vega & Kaya, 2012). The role of *P. fuscipes* originating *Pseudomonas* strains needs to be disclosed in future studies. Our designed species‐specific *16S rRNA *primers could identify only *Pseudomonas*‐like PPB (Figure 2), an advantage that will be useful for the determination of PPB circulation pattern in the life cycle of *Paederus* beetles.

To raise the sensitivity and specificity of *Wolbachia* DNA amplification, we used a nested‐PCR assay (Karami et al., 2016). Generally, in many specimens, PCR products of the first step were positive; however, in a few cases, the density of *Wolbachia* indeed was so low (as indicated by Arthofer, Riegler, Avtzis, & Stauffer, 2009) that we have to perform the second step. The use of other techniques, including high‐quality polymerases, amplicon detection via DNA probes (Arthofer, Riegler, Schneider, et al., 2009) or high‐throughput sequencing methods (NGS), is recommended. The frequency of *Wolbachia* in 128 species of beetles belonging to seven families of Buprestidae, Hydraenidae, Dytiscidae, Hydrophilidae, Gyrinidae, Haliplidae, and Noteridae showed to be 31% (Sontowski, Bernhard, Bleidorn, Schlegel, & Gerth, 2015). Oliveira et al. (2015) used three markers (*16S rRNA*, *wsp*, and *ftsZ*) to screen a broad range of Brazilian insect species and found *Wolbachia *infection in 13% (*n* = 25) of the studied coleopterans (Oliveira et al., 2015). Infection of *P. fuscipes* by *Wolbachia* strains was originally reported by Yun et al., (2011). They did not track the prevalence of *Wolbachia* infection in the rove beetles but provided evidence for indirect horizontal transmission of *Wolbachia* between predators and preys (Yun et al., 2011). In the present study, *Wolbachia* (combined) infection rate in female and male specimens was 88.23% and 67.57%, respectively (χ^2^, *p *= 0.015). This difference is remarkable as the infection rates are in accordance with other studied insects including mosquitoes (Karami et al., 2016), and the fact is that no study has already been compared *Wolbachia *infection rates in the male and female beetles.

Herein, the phylogeny of *P. fuscipes*‐infecting *Wolbachia* was not investigated; nonetheless, they were previously classified in the supergroup B, based on the *16S rRNA* and *wsp *markers (Yun et al., 2011). MLST data are needed to determine their exact position among 16 supergroups.

Surprisingly, the coinfection rates of both PPB and *Wolbachia *were 70.59% in females and 17.57% in males. The frequency of both bacteria in females was four times that of males (χ^2^, *p* < 0.0001). This co‐occurrence may imply putative interactions among these endosymbionts.

Our results highlighted the coexistence of PPB (as defensive) and *Wolbachia* (as reproductive) secondary endosymbionts not only in females but also in males of *P. fuscipes*. These bacteria will potentially interact with the host beetle and with each other as well. As defined in defensive symbiosis, the symbionts protect their host against hostile agents, including pathogens, parasites, parasitoids, or predators by the production of diverse metabolites, antimicrobial compounds, or toxins (Flórez, Biedermann, Engl, & Kaltenpoth, 2015). Defensive compounds such as pederin, piericidin, streptochlorin, and diaphorin have been characterized from bacterial symbionts of diverse insects (Beemelmanns, Gio, Rischer, & Poulsen, 2016). Although pederin can protect *Paederu*s species from predation by natural enemies (Kellner & Dettner, 1995, 1996), its protective role against parasitoid wasps or entomopathogenic nematodes has not been inspected (Oliver & Moran, 2009). Also, the effects of *Wolbachia* infection on the life history of *Paederus* spp. are unclear. The reproductive phenotypes caused by *Wolbachia* in the *P. fuscipes* will need to be determined in the future surveys.

Given the transovarial transmission of *Wolbachia* as well as its relation to the reproductive phenotypes, the attention of researchers on *Wolbachia* infections should be drawn to the reproductive tissues. Dobson et al. (1999) have conversely demonstrated that *Wolbachia *infections not only are distributed in germ line but also are present throughout insect somatic tissues. They have also reported that the distribution of *Wolbachia* in somatic tissues is varied between different *Wolbachia*/host associations (Dobson et al., 1999). Distribution of *Wolbachia* in the somatic and reproductive tissues of *Paederus* species needs to be determined in future.

The interaction between the PPB and *Wolbachia* has not been studied in any case. However, the asymmetrical interaction of *Wolbachia* and *Spiroplasma* endosymbionts had been indicated in the *Drosophila melanogaster* by Goto, Anbutsu, and Fukatsu (2006) who showed that *Wolbachia* could not affect the population of *Spiroplasma*, while *Spiroplasma* could negatively restrict the population of *Wolbachia*. Remarkably, they could not detect *Wolbachia* from the fly hemolymph, the principal location of *Spiroplasma *(Goto et al., 2006). Insect hemolymph is an operational area for innate immune responses where the phenol oxidase cascade factors, antimicrobial peptides, phagocytosis, and encapsulation of exotic agents are produced by hemocytes (Lavine & Strand, 2002; Naitza & Ligoxygakis, 2004; Theopold, Li, Fabbri, Scherfer, & Schmidt, 2002). In *Paederus* beetles, the addition of pederin toxin to the hostile environment of the hemolymph may render the condition more difficult for dwelling microorganisms, requiring further investigation.

Our results reported more frequency of both bacteria in females than that of males (χ^2^, *p* < 0.0001). This observation may indicate tripartite interactions among *Paederus*, *Wolbachia,* and PPB. Recently, it has been proposed that the nature of the interaction between the insect host and *Wolbachia* bacterium is parasitic or mutualistic, and the induction/inhibition of reactive oxygen species would be an essential player in the new and native hosts (Zug & Hammerstein, 2015). The nature of *Paederus*–*Wolbachia* interaction is not known and requires being determined in upcoming studies. Moreover, it has previously been reported that antimicrobial peptides keep the insect's endosymbionts under governor (Login et al., 2011). It is unclear whether the PPB regulates the population of *Wolbachia* via pederin or not. Hence, co‐occurrence of *Wolbachia* and PPB in rove beetles may infer that *Wolbachia* is adapted to cope with adverse conditions triggered by PPB. Numerous *Wolbachia* strains have already been found in beetle's eggs containing antimicrobially active components (Pankewitz, Zollmer, Hilker, & Graser, 2007). Thus, it seems that these kinds of adaptations are common features among the *Wolbachia* strains. As a conclusion, on the side of symbiosis, PPB and *Wolbachia* may interact with each other and *Paederus* beetles, while on the side of insect host, *Paederus* beetles exploit these defensive and reproductive symbionts to warrant their fitness in the environment. Details and nature of these interactions (even at gender level) call for further investigation and testing.

## CONFLICT OF INTEREST

The authors declare that there is no conflict of interest.

## AUTHORS CONTRIBUTION

NDD, NMR, and AAR conceived and designed the experiments. NMR and MJ collected the samples. NA performed the molecular experiments. NMR wrote the paper. NMR and AAR went through bioinformatics analyses. NMR and NDD analyzed and interpreted total data. NMR, NDD,SZ, and AAR involved in critical revision of manuscript. NDD and SZ financially supported the research. All authors read, discussed the results, and contributed to the final version of manuscript.

## ETHICS STATEMENT

None required.

## Data Availability

All data are included in the main manuscript. Sequences were also have been deposited at the NCBI GenBank under accession number of KY568928–KY568936, KY568937–KY568941, and KY555600–KY555603.
